# BCFtools/RoH: a hidden Markov model approach for detecting autozygosity from next-generation sequencing data

**DOI:** 10.1093/bioinformatics/btw044

**Published:** 2016-01-30

**Authors:** Vagheesh Narasimhan, Petr Danecek, Aylwyn Scally, Yali Xue, Chris Tyler-Smith, Richard Durbin

**Affiliations:** 1 ^1^Wellcome Trust Sanger Institute, Hinxton and; 2 ^2^Department of Genetics, University of Cambridge, Cambridge, UK

## Abstract

**Summary:** Runs of homozygosity (RoHs) are genomic stretches of a diploid genome that show identical alleles on both chromosomes. Longer RoHs are unlikely to have arisen by chance but are likely to denote autozygosity, whereby both copies of the genome descend from the same recent ancestor. Early tools to detect RoH used genotype array data, but substantially more information is available from sequencing data. Here, we present and evaluate BCFtools/RoH, an extension to the BCFtools software package, that detects regions of autozygosity in sequencing data, in particular exome data, using a hidden Markov model. By applying it to simulated data and real data from the 1000 Genomes Project we estimate its accuracy and show that it has higher sensitivity and specificity than existing methods under a range of sequencing error rates and levels of autozygosity.

**Availability and implementation**: BCFtools/RoH and its associated binary/source files are freely available from https://github.com/samtools/BCFtools.

**Contact:** vn2@sanger.ac.uk or pd3@sanger.ac.uk

**Supplementary information:** Supplementary data are available at *Bioinformatics* online.

## 1 Introduction

The prevalence of runs of homozygosity (RoHs) varies worldwide depending on past demography and recent mating patterns. Targeted studies in families from populations with high consanguinity have been successful in identifying genes underlying simple autosomal recessive disorders using autozygosity mapping. While several methods exist to detect these RoH (Browning and Browning, 2010; Gusev *et al*., 2009; Purcell *et al*., 2007) they were primarily designed for genotyping data and do not exploit all the information available from population sequencing, which includes fuller information about allele frequencies and recombination rates; moreover, they were not designed to be computationally efficient to accommodate the higher complexity of sequence data. More recently, Magi *et al*. (2014) introduce a new method H3M2 developed for sequence data but which requires BAM files, which are not always available. We present a software package, BCFtools/RoH, to allow geneticists carrying out genome-wide sequencing studies to infer autozygous sections from sequence-derived variation data in a more accurate and more efficient way.

## 2 Methods

### 2.1 Identifying autozygous sections of diploid genomes using a hidden Markov model

BCFtools/RoH uses a hidden Markov model (HMM) to identify ROHs. The HMM is applied to genetic variation data (in VCF format) for the population containing the sample, with positions in the chain corresponding to segregating sites in the population, and using either genotype calls or genotype likelihoods. The two hidden states represent extended homozygosity (H) and non-homozygosity (N) within the sample. Genotypes are represented by RR for a homozygous site matching the reference, RA for a heterozygous site and AA for a homozygous alternate (non-reference) site. Thus, H tracts can only include RR and AA sites, whereas N tracts can include sites of any genotype.

Emission probabilities in N regions correspond to a Hardy–Weinberg model, and thus for any site *i* are determined by the minor allele frequency *f_i_* at that site (excluding non-biallelic sites) and likelihoods of observed alignment data for possible genotypes in the sample (provided by the variant calling algorithm):

$$P\left(D_{i}|X_{i}=H\right)=\left(1–f_{i}\right)P\left(D_{i}|RR\right)+f_{i}P\left(D_{i}|AA\right)$$

$$P\left(D_{i}|X_{i}=N\right)={\left(1–f_{i}\right)}^{2}P\left(D_{i}|RR\right)+2f_{i}\left(1–f_{i}\right)P\left(D_{i}|RA\right)+f_{i}{}^{2}P\left(D_{i}|AA\right),$$

where *D_i_* represents the data (i.e. aligned reads) and *X_i_* is the homozygosity (hidden) state at site *i*. When utilizing genotype calls instead of likelihoods, we allow the user to specify the confidence in the calls on the Phred scale, and these are then converted and used as genotype likelihoods with the specified level of error (confidence) for each genotype. To account for gaps of missing data between sites (due to the use of exome data, for example) the transition probabilities in the HMM incorporate the likelihood of a recombination event since the last site. We obtain *ρ_i_*, the recombination rate at site *i*, by interpolating the fine scaled genetic map between positions *i + 1* and *i* (Kong *et al.*, 2010) and allowing for only a single recombination event between those positions. This is then multiplied by the probabilities for transitions between states, *p*_NH_ and *p*_HN_.

$$P\left(X_{i}{}_{+1}=N|X_{i}=N\right)=\;1–\rho_{i}p_{\text{NH}}$$

$$P\left(X_{i}{}_{+1}=H|X_{i}=N\right)=\rho_{i}p_{\text{NH}}$$

$$P\left(X_{i}{}_{+1}=N|X_{i}=H\right)=\rho_{i}p_{\text{HN}}$$

$$P\left(X_{i}{}_{+1}=H|X_{i}=H\right)=\;1–\rho_{i}p_{\text{HN}}$$

The parameters *p*_NH_ and *p*_HN_ are learnt from the data using a Viterbi training scheme (Durbin *et al.*, 1998). For the initial probability of being in the *H* state at the start of each chromosome, we used the inbreeding coefficient estimated for each individual, calculated using a method of moments estimator. The resulting state assignments given by the Viterbi sequence with the optimized parameters comprise our inferred RoH and non-RoH tracts.

## 3 Results

### 3.1 Validation of the method on simulated data

**Fig. 1.**
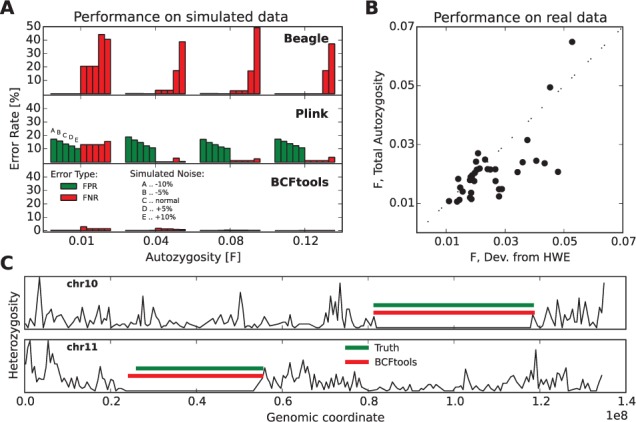
Comparison of error rates of BCFtools/RoH and other existing methods as well as performance on real data. (**A**) Performance on simulated data. FPR and FNRs in data simulated with varying levels of autozygosity and SNP calling error, analyzed using three different detection methods. (**B**) Performance on real data. We compare the inbreeding coefficient *F*, estimated either by our method as the percentage of the genome that is autozygous, or as the deviation from HWE estimated across all sites, for 31 CEU individuals. (**C**) Example autozygous segments in simulated data (green) and detected by our method (red). For each chromosome, the *y*-axis shows the normalized density of heterozygous sites in bins of 0.1 Mb. The *x*-axis shows the position of the chromosome (in units of 1e8 bp). The overlapping red and green sections show that the regions identified as autozygous using our HMM approach accurately reflect the true length and location of autozygous sections in the simulated data

In order to test our model against a dataset for which the autozygous states are known, we simulated sequence variation data from exome capture regions using a Markov process for which we varied the expected number and segment length of autozygous sections in 10 linear steps ranging from 50 (half sibling mating) to 2 Mb (MRCA ∼20 generations ago), giving overall autozygosity between 12 and 1%. As SNP calls may have errors, and in order to test our model’s robustness to this, in some simulations we randomly added 10 and 5% more heterozygous positions to the sequence generated by the standard simulation to reflect false positive error rates seen in both low and high coverage real sequence data. Similarly, we uniformly changed 5 and 10% of heterozygous positions to homozygous in the sequence in other simulations. Allele frequency and variant position information from 99 CEU samples from the 1000 Genomes Project (Abecasis *et al*., 2012) as well as information on the human recombination rate from linkage studies (Kong *et al*., 2010) were used to produce a test dataset that included 1 130 894 SNPs. In total, 110 simulated datasets with different parameters were generated. The simulated datasets were then run through our inference process. When compared against true autozygous sites (see Supplementary methods), the mean false positive rate (FPR) and false negative rate (FNR) across all simulations were 0.04 and 0.83%, respectively, with maximum values of 0.18 and 3.03% (Fig. 1A, Supplementary Fig. S1). Further, the output of our model was compared with the true regions and shows a close match (Fig. 1C). We also examined the effects of downsampling the data in terms of samples and sites and showed that sites discovered to be autozygous did not change from the true regions by more than 20% (Supplementary Figs S2 and S3).

### 3.2 Comparison of the method to existing approaches that detect RoHs from VCF files

In order to compare our approach to existing methods, we ran two complementary approaches on our simulated data that have either been used in previous RoH studies or were the focus of a recent publication on detection of autozygosity (Howrigan *et al*., 2011), using the default settings. For the range of autozygosity and SNP calling error in our simulations above, we calculated the FPR and FNR (see Section 2) and found that our approach showed the lowest error rates (Fig. 1A). Beagle performs similarly to our method given no introduction of SNP ascertainment error but is not robust to the addition of random heterozygote genotypes. Plink, due to its windowing approach, consistently overestimates the size of a region and has an FPR of >10% on all simulations. We note that H3M2 also was reported to give similar performance, based on simulated exome BAM files.

### 3.3 Application of the method to real data from the 1000 Genomes Project

We applied our method to 2504 individuals from Phase 3 of the 1000 Genomes Project, both running on the full genome VCFs and on the exomes. The fraction of the genome found to be autozygous varies by individual and population (Supplementary Fig. S4). The exome results provide estimates that are within 5% of the whole genome estimate (Supplementary Fig. S5). Our estimates are also close to independent estimates of the total percentage of autozygosity measured by the inbreeding coefficient, in 31 individuals for which it was at least 1% (Fig. 1B).

## Funding

This work was supported by Wellcome Trust grants WT099769 (V.N.), and WT098051 (C.T.-S., P.D., R.D., Y.X.).

*Conflict of Interest:* none declared.

## Supplementary Material

Supplementary Data
