# Supplementary material for: BCFtools/RoH: a hidden Markov model approach for detecting autozygosity from next-generation sequencing data
Source: Bioinformatics. 2016 Jan 30;32(11):1749–51. doi: 10.1093/bioinformatics/btw044 (PMC4892413; doi:10.1093/bioinformatics/btw044)
Supplement: Supplementary Data [file supp_32_11_1749__index.html]

BCFtools/RoH: a hidden Markov model approach for detecting autozygosity from next-generation sequencing data — Supplementary Data 

# BCFtools/RoH: a hidden Markov model approach for detecting autozygosity from next-generation sequencing data

## Supplementary Data

files

- Supplementary Data - docx file
