# Supplementary material for: BCFtools/RoH: a hidden Markov model approach for detecting autozygosity from next-generation sequencing data
Source: Bioinformatics. 2016 Jan 30;32(11):1749–51. doi: 10.1093/bioinformatics/btw044 (PMC4892413; doi:10.1093/bioinformatics/btw044)
Supplement: Supplementary Data [file supp_btw044_Appnote_Supplementary_Information.docx]

**Supplementary Information**

**Pre-processing of real and simulated data**

For each variable site, we calculated the minor allele frequency as well as the distance between that site and the previous site. In simulated data, sequencing errors at the error rates described were introduced after the data were generated. Missingness at a site was handled by dropping the site entirely. Heterozygosity estimates to initialize the hidden Markov model were first calculated for each sample by taking the expected deviation from Hardy-Weinberg equilibrium across all sites (see below),

**Calculation of FPR/FNR in simulated data**

For each run of the data with the specified parameters, we obtain the difference in the calls of the detected autozygous segments and its corresponding contiguous segment in the simulated data. We then classified a site as a False Positive if it is called in our inference but is non-autozygous in the simulated data. Similarly, the False Negatives are sites that are autozygous in our simulation but not in the inference. For each sample, we calculated the false positive rate as the number of incorrectly called sites where the inferred sample calls a segment as autozygous when none is present, divided by the total number of simulated non-autozygous sites. The false negative rate was the total number of false negatives divided by the total number of sites that are actually autozygous. As an example, for a sample with autozygosity of 0.062 and genotyping error rate 0.05%, Table S1 gives the FPR and FNR.

|  |  | Simulated (Truth) | |
| --- | --- | --- | --- |
|  |  | 1 | 0 |
| Inferred (Test) | 1 | True Positive (TP) = 28345 | False Positive (FP) = 101 |
|  | 0 | False Negative (FN) = 331 | True Negative (TN) = 434613 |
|  |  | FNR = FN / (TP + FN) = 1.15% | FPR = FP / (FP + TN) = 0.023% |

**Table S1 Error Rates on inference on a single simulated sample**

Comparison of a site being autozygous on the truth dataset as compared with the test data. Each of the 4 configurations of simulation and inference are classified as true and false, positives and negatives and the FPR and FNR are calculated.

**Calculation of inbreeding coefficient estimates**

In order to compare the overall autozygosity we estimate in samples from the 1000 Genomes Project, we first computed the deviation in allele frequency estimates due to inbreeding(Wigginton, Cutler, & Abecasis, 2005). Here, we used a method of moments estimator where we obtained the deviation from Hardy-Weinberg equilibrium that occurs due to inbreeding. A large proportion of this deviation will occur due to the contribution of sites from autozygous sections. As such, we can compare this estimate with our estimate of the total region that is predicted to be autozygous to see that if are similar. Specifically, for each individual, we obtained the total number of genotyped loci *N*, the total number of observed homozygous loci O, and the total number of loci that are expected to be homozygous *E* and calculated the inbreeding coefficient *F* as *F = (O-E)/(N-E)*. This was then compared to the number of autozygous sites detected divided by the total number of variant sites in the individual sample, *F_roh_*. We demonstrate that this would serve as an independent manner to validate our performance on real data by reporting the concordance of the inferred autozygosity on simulated data with that obtained from this estimator, (Pearson correlation = 0.971, p-value <2.2e-16).


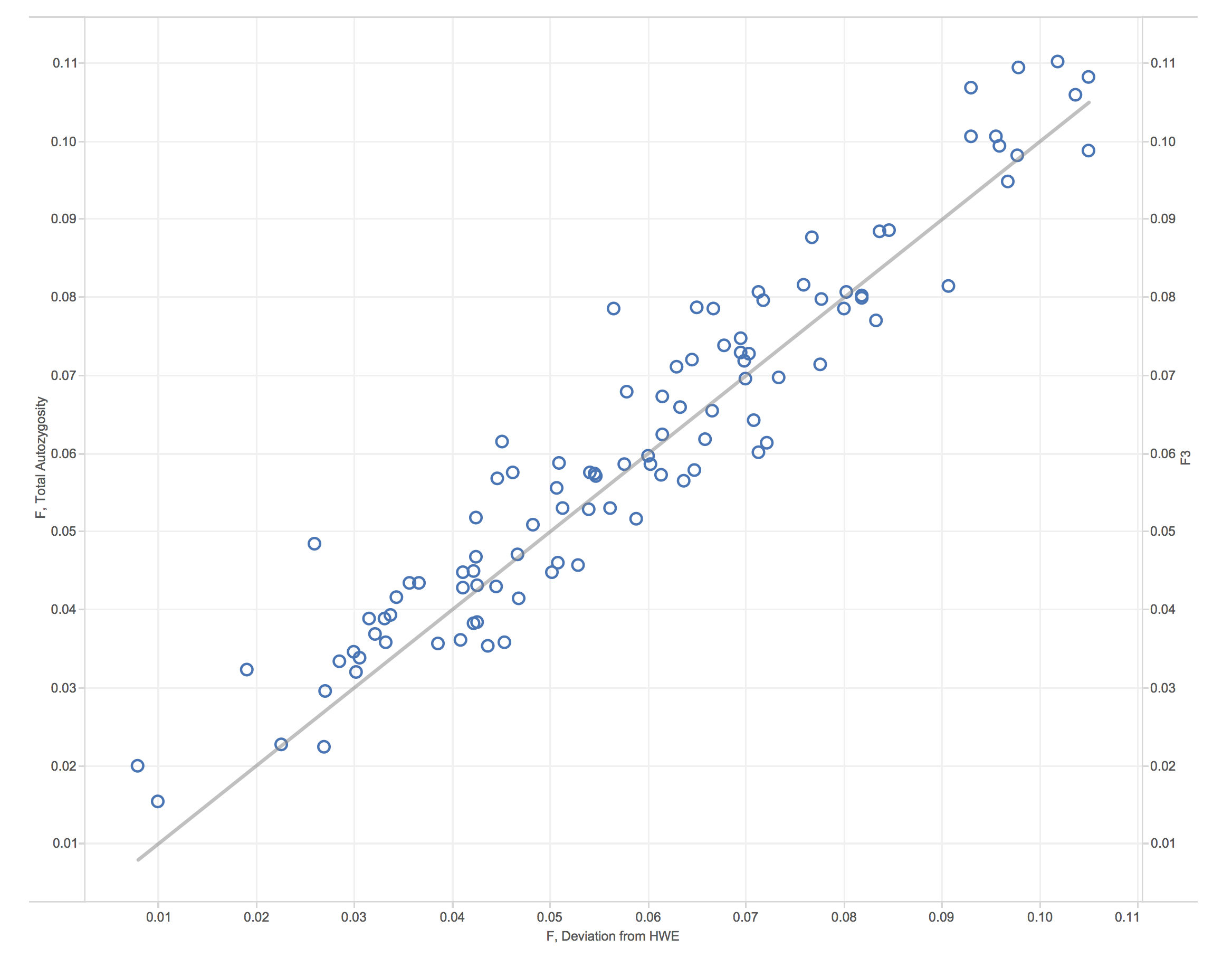


**Figure S1: Correlation between the method of moments estimator and inbreeding coefficient as calculated using autozygosity from simulated sequence data**

The two measures of estimating the inbreeding coefficient are highly correlated and can be used as independent assessments of the measure of autozygosity in an individual.

**Simulation of exome data and creation of test dataset**

In this section we describe in detail the process by which the simulated genomes were created for the inference procedure. We start by obtaining allele frequency and site information from data from the 1000 genomes project. We restricted our analysis to 99 individuals from the CEU population from Phase I of the project and sites that were within the exome capture baits. We then simulated a markov process that transitioned between two states, extended homozygosity (H) and non-homozygosity (NH) as in BCFtools ROH. When in the NH state, the markov model would emit genotypes RR, RA and AA (R – Reference, A – Alternate) at each particular site according to the allele frequency and Hardy-Weinberg equilibrium. When in the H state, the model only emits the RR and AA genotypes with probability *f* and *1-f* respectively, where *f* is the allele frequency. The transition matrices in the Markov process were chosen to reflect the extent of autozygosity in individuals with varying degrees of parental relatedness in terms of (a) the number of segments observed and (b) the length of such segments. Individuals with high amounts of autozygosity are expected to come from close unions and will have segment lengths that are typically longer as well as many more such segments. To capture this, we increased the transition probabilities from the NH state to the H state and decreased the transition probability from the H state to the NH state in ten linear steps that reflects expected segment lengths from 50Mb (half sibling mating) to 2Mb (MRCA ~20 generations ago), and overall autozygosity rates between 12% and 1%. Additionally, we utilized the fine-scale human recombination map to vary the transition probabilities according the frequency of recombination observed in real data. Once we obtained a set of sequence data with generated genotypes according to our model above, we wanted to assess the effect of introducing SNP errors on the detection of RoH segments by the inference procedure. As all RoH algorithms ultimately rely on the density of homozygote sites seen in the data we wanted to assess the effect of removing or adding in such regions to reflect two scenarios that cover false positive and false negative rates in real genetic data. To simulate false positives, we distributed an additional 5% and 10% more heterozygotes randomly genome wide. To simulate false negatives we changed 5% and 10% of heterozygous mutations to homozygous.

**Estimating the probability of recombination between positions**

In our algorithm, we account for potential recombination events between a given pair of contiguous variants by estimating the probability of recombination based on the genetic map positions of the variants. For each position *i*, we obtain the genetic map coordinates at position i, from the fine-scaled recombination map (Kong et al., 2010). As the genetic map does not define coordinates for every physical location in the genome, we linearly interpolate the genetic map position between the two closest positions defined on the genetic map. We then obtain the rate ρ_i_, the probability of recombination since the last site by taking the difference in the genetic map coordinates g_i_ and g_i+1_ and obtain a rate in terms of the physical distance d_i_ between the SNPs as $\frac{g_{i+1}-g_{i}}{d_{i+1}-d_{i}}$. We note that through this process we only allow for only a single recombination event between those positions regardless of the length of the interval between the site of interest and the previous site.

**Effect of allele frequency accuracy on RoH detection**

We wanted to assess the effect of decreasing our accuracy in measuring the allele frequency information of a population on the autozygosity measurement, by simulation. In order to do this, we subsampled the 2625 individuals from Phase 3 of the 1000 Genomes Project (Auton et al., 2015) at various counts, calculated the allele frequency information at each site and ran our inference with the subsampled allele frequencies at those sites on a single individual with 10% autozygosity. As the subsampled individuals were randomly selected, we performed the same simulation 10 times, to adjust for this stochasticity in the measurement, and obtained the average error rates across the 10 different simulations. To ensure that our error rates was as conservative as possible and to account for population effects we assessed our errors on the set of sites that were obtained from our entire dataset of 2625 individuals. We call a site for which we don’t have data as lying in an autozygous stretch if the sites preceding and after it also lie in one. We see that as we reduce the number of individuals in our dataset, both our false positive and negative rate increase.

**Figure S2: Effect of reducing the number of individuals on error rates**

Points represent the mean error rates (FPR in orange and FNR in blue) on an individual with 10% overall autozygosity. The X-axis represents the number of individuals used to calculate allele frequency information. The left and right hand y-axes represent the FPR and FNR respectively.

**Effect of down sampling/missing data on the FP and FN Rates**

We wanted to examine the effect of missing/sparse data might have on the error rates of the model. In order to explore this, we simulated data for a single individual with 10% autozygosity and subsampled random sites in the genome in order to retain between 50% to 100% of all our original sites from the 1000 Genomes Project reference upon which the synthetic genomes are constructed. We then performed our inference on the remaining sites and calculated the error rates as we had done previously by evaluating the called regions over the true regions simulated from a dataset containing all of the original variable sites. We note that a reduction in sites has a minimal effect on the FPR but we begin to undercall true autozygous regions as we reduce our site density.

**Figure S3: Effect of reducing the number of sites on error rates**

The X-axis reflects the percentage of sites that remain after subsampling. The FPR is indicated by points in orange with values given by the left hand Y-axis. The FNR is indicated by points in blue measured according to the right hand Y-axis.

**Application of our model on real data from the 1000 genomes project**

We downloaded the integrated low coverage whole genome, Agilent exome and Illumina Omni genotyping data from Phase 3 of the 1000 Genomes Project, and considered only bi-allelic SNP sites. These were then processed by calculating the allele frequencies estimated using only individuals from each of the specific populations. Then we ran BCFtools/RoH with the following settings:

bcftools roh -b -e subset population.txt -G30 -I -a 6.6e-08 -H 5e-09 -v

To characterize the total autozygosity for each population, we counted the total number of callable bases in the autozygous sections and divided that by the total number of callable bases in the genome. We define callable regions as those that lie within the 1000 genomes accessibility mask. We note that some of the South Asian populations have the highest amounts of autozygosity amongst 1000 Genomes Project populations which is in line with higher levels of endogamy and population structure within those populations. Within Europe, the Finnish population, which is known to have a second population bottleneck, has a higher level of autozygosity in comparison with the others.

**Figure S4: Application of our method to real data from Phase 3 of the 1000 Genomes Project**

The distribution of levels of autozygosity per population are given in boxplots. Outliers for each of the populations are in colored dashes and boxplots are ordered by mean autozygosity in each continental group

**Comparison of our model on exome and whole genome low coverage data on the same set of samples**

In order to see if our tool could be applied to datasets from whole genome and whole exome data with different properties of site choice and allele frequency, we compared the results of our model on a set of samples that was captured on both platforms. We created an exome dataset by restricting the 1000 Genomes Project phase I dataset to just the exome sites and compared the autozygous segments obtained with those from the original whole genome data. To compare the two datasets without biases towards regions that might be undetectable because they lie outside of the exome bait regions, we restricted the comparison to segments at least 500kb long. We show that the two estimates are highly correlated (gradient = 1.057, p<0.0001) and that the exome estimates are on average 0.14% lower than those from whole genome, due to missing data at the ends of autozygous runs.

To further characterize the difference between the exome and whole genome inferences in terms of RoH length, we used a definition provided by (Pemberton et al., 2012; Szpiech et al., 2013). They define, short ROH (class A) as those that are tens of kilobases in size and reflect homozygosity of ancient haplotypes that predate continental migrations, medium ROH (class B) as those that are hundreds of kilobases to a few megabases long and mostly arise from background relatedness within populations and finally long ROH (class C) as those that are several megabases long and probably result from recent parental relatedness. Here we defined these classes as follows:

Class A – short ROH – length <500kb

Class A – medium ROH – 500kb < length <2Mb

Class C – long ROH – length > 2Mb

To compare our results across the various RoH classes we adopt an approach similar to one used (Pippucci, Magi, Gialluisi, & Romeo, 2014) by examining the total length of RoH in each class across data from the 1000 genomes project. We chose to show the comparison on a per individual basis for clarity. We see that for RoH of class A and B we undercall the mean amount of RoH present in segments of that size perhaps because they lie in regions that are not covered by the exome baits. For large regions such as those in class C we were limited by the data we had and there were fewer than 20 samples with regions of such length. Given this limitation, for larger regions such as those in class C we see that we over-estimate the length of such segments probably owing to the fact that the transition to the non-homozygous state happens at the next possible SNP position in exome data which might be quite some distance from the actual recombination event.

**
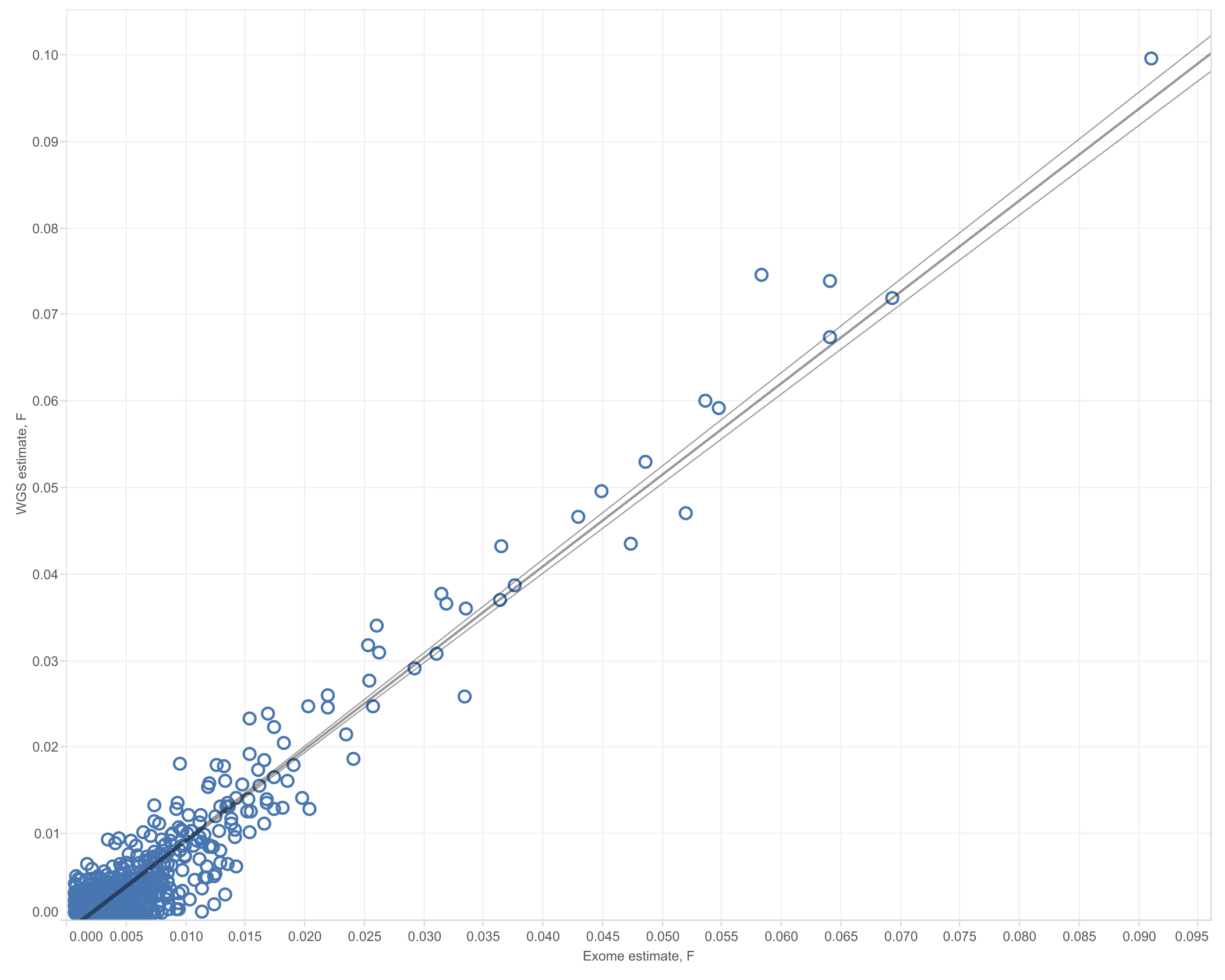
**

**Figure S5: Application of our model to WGS and exome data**

****Graph showing the estimates for total autozygosity from samples obtained from whole genome and exome sequences from phase 1 of the 1000 genomes project show that exome data is effective for discovering autozygous stretches within 1% of the genome-wide value. The regression line across the data along with its confidence interval is reflected in grey lines.

**Figure S6** Comparison of ROH detection on length of certain class (A, B and C) and on whole genome (wgs) and exome datasets

**Comparison of our model on simulated whole genome data**

We obtain whole genome data from chromosome 10 of the 1000 Genomes Project. Similar to analysis with the exomes, we restricted our analysis to 99 individuals from the CEU population from Phase I of the project. We then simulated data for genotypes in the same manner as described above in the section titled (Simulation of exome data and creation of test dataset) and compare the performance of our method against those from the other methods (Figure S7). We find that our error rates between whole genome and exome datasets are comparable: both the exome and whole genomes datasets have False Positive and False Negative rates under 10%. For whole genome data beagle performs similarly to bcftools (in contrast to exome data where it as a higher false negative rate) whereas has higher false positive rates.


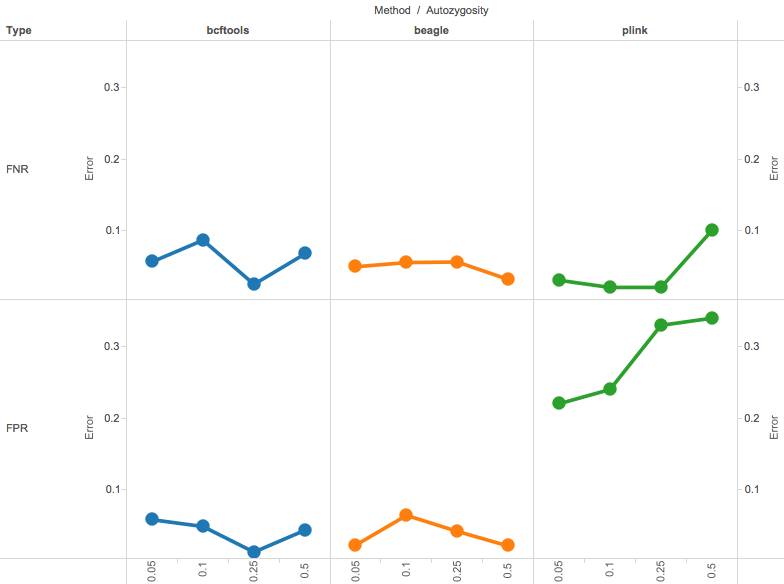


**Figure S7** Comparison of various methods on test data from whole genome sequences show that BCFtools ROH has FP and FN rates that are under 10% across a range of simulated scenarios, which is comparable in performance to beagle. Both methods outperform plink which works on windows of fixed length

**Comparison of method using constant transition parameters across a range of autozygosity**

In order to test our model to it’s robustness towards the choice of transition parameters we chose to keep these parameters (*p*_NH_ and *p*_HN_) constant at 6.67x10^-6^ and 5x10^-6^ and vary the amount of autozygosity observed in the sample. Error rates remain under 2% regardless of the amount of autozygosity in the sample, indicating that inference of state is dominated by the emission probabilities (Figure S7). As the range of autozygosity simulated represents a range of pedigrees, we note that the method is robust to our initial assumption of having transition rates that are expected for the offspring of first cousins (our initialization).


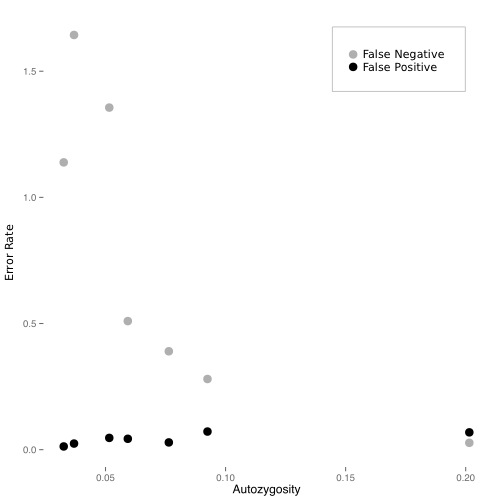
**Figure S8** Comparison of false positive and negative rates with differing amounts of autozygosity keeping *p*_NH_ and *p*_HN_ constant.

**REFERENCES**

Auton, A., Abecasis, G. R., Altshuler, D. M., Durbin, R. M., Bentley, D. R., Chakravarti, A., … Schloss, J. A. (2015). A global reference for human genetic variation. *Nature*, *526*(7571), 68–74. http://doi.org/10.1038/nature15393

Kong, A., Thorleifsson, G., Gudbjartsson, D. F., Masson, G., Sigurdsson, A., Jonasdottir, A., … Stefansson, K. (2010). Fine-scale recombination rate differences between sexes, populations and individuals. *Nature*, *467*(7319), 1099–103. http://doi.org/10.1038/nature09525

Pemberton, T. J., Absher, D., Feldman, M. W., Myers, R. M., Rosenberg, N. A., & Li, J. Z. (2012). Genomic patterns of homozygosity in worldwide human populations. *American Journal of Human Genetics*, *91*(2), 275–92. http://doi.org/10.1016/j.ajhg.2012.06.014

Pippucci, T., Magi, A., Gialluisi, A., & Romeo, G. (2014). Detection of runs of homozygosity from whole exome sequencing data: state of the art and perspectives for clinical, population and epidemiological studies. *Human Heredity*, *77*(1-4), 63–72. http://doi.org/10.1159/000362412

Szpiech, Z. A., Xu, J., Pemberton, T. J., Peng, W., Zöllner, S., Rosenberg, N. A., & Li, J. Z. (2013). Long Runs of Homozygosity Are Enriched for Deleterious Variation. *The American Journal of Human Genetics*, *93*(1), 90–102. http://doi.org/10.1016/j.ajhg.2013.05.003

Wigginton, J. E., Cutler, D. J., & Abecasis, G. R. (2005). A note on exact tests of Hardy-Weinberg equilibrium. *American Journal of Human Genetics*, *76*(5), 887–93. http://doi.org/10.1086/429864
